# Dietary patterns in relation to glioma: a case–control study

**DOI:** 10.1186/s40170-024-00336-4

**Published:** 2024-03-18

**Authors:** Mohammad Nemati, Mehdi Shayanfar, Fatemeh Almasi, Minoo Mohammad-Shirazi, Giuve Sharifi, Azadeh Aminianfar, Ahmad Esmaillzadeh

**Affiliations:** 1 Department of Community Nutrition, School of Nutritional Sciences and Dietetics, Tehran University of Medical Sciences, Tehran, Iran; 2 Department of Clinical Nutrition and Dietetics, National Nutrition and Food Technology Research Institute, Shahid Beheshti University of Medical Sciences, Tehran, Iran; 3 Research Center for Biochemistry and Nutrition in Metabolic Diseases, Institute for Basic Science, Kashan University of Medical Sciences, No. 226, Ravand Blv, Kashan, 1416753955 Iran; 4 Department of Neurosurgery, Loghman Hakim Hospital, Tehran, Iran; 5 Obesity and Eating Habits Research Center, Endocrinology and Metabolism Molecular-Cellular Sciences Institute, Tehran University of Medical Sciences, Tehran, Iran; 6 Department of Community Nutrition, School of Nutrition and Food Science, Isfahan University of Medical Sciences, Isfahan, Iran

**Keywords:** Glioma, Cancer, Brain tumor, Diet, Dietary pattern, High protein, Vegetarian, Western, Case–control

## Abstract

Although the association of individual foods and nutrients with glioma have been investigated, studies on the association of major dietary patterns and glioma are scarce. The aim of this study was to examine the association between major dietary patterns and risk of glioma in a group of Iranian adults. In this hospital-based case–control design, we recruited 128 newly diagnosed glioma cases and 256 controls in Tehran from 2009 to 2011. A Willett-format-validated 126-item semi-quantitative Food Frequency Questionnaire (FFQ) was used to assess participants' dietary intake. Factor analysis was used to identify major dietary patterns. We identified 3 major dietary patterns using factor analysis: high protein, vegetarian and western dietary pattern. After several adjustments for potential confounders, adherence to the high protein dietary pattern was inversely associated with risk of glioma (OR: 0.47; 95% CI: 0.23, 0.95). Consumption of vegetarian dietary pattern was also associated with a reduced risk of glioma (OR: 0.16; 95% CI: 0.07, 0.34). Greater adherence to the western dietary pattern was associated with a greater chance of glioma (OR: 3.30; 95% CI: 1.52, 7.17). We found that high protein, vegetarian and western dietary pattern were significantly associated with glioma risk. Further prospective studies are needed to confirm these findings.

## Introduction

Cancers are the leading cause of premature death in developed countries [1]. Glioma is the most common type of brain cancer, accounting for nearly 80% of all malignant primary intracranial tumors [2]. It is often diagnosed in advanced stage and is poorly responsive to currently available treatments [3], therefore, median overall survival of patients with glioma is less than 2 years [4]. In 2019, a total of 296,851 new cases of cancers of the brain and central nervous system were diagnosed [1]. In Iran, incident primary brain tumors is shown as 5.69 per 100,000 person-years by a significant gender difference; such that it was prevalent among men than women [5].

The relationship between diet and adult glioma has been the focus of nutritional investigations in recent years [6]. Although diet-disease associations can be explored in terms of individual nutrients and foods, most nutritional epidemiologists suggested the approach of dietary patterns when investigation such associations [7]. Assessment of individual nutrients and foods have several limitations which could be overcome by looking at dietary patterns [7]. Although dietary patterns have been examined in relation to several health-related outcomes including cancers [8–11], few studies are available assessing the link between major dietary patterns and glioma. In a case–control study, the association between dietary patterns during adolescence and the occurrence of brain tumors in adulthood was investigated. The researchers of that study suggested that a dietary patterns high in sugar and fat-rich foods was associated with a lower probability of developing intracranial tumors [12]. In a combined analysis of 3 large prospective studies in the UK and US revealed a weak association between adherence to healthy dietary patterns, as measured by the dietary approaches to stop hypertension (DASH) eating plan, alternate Mediterranean diet and Alternative Healthy Eating Index (AHEI), and increased risk of glioma; however, these associations were generally null after excluding the first 5 years of follow-up [13]. The findings of a meta-analysis showed that following a healthy diet and consuming more vegetables and fruits was associated with reduced risk of glioma, but the findings regarding the consumption of whole grains, fresh fish, dairy products and nuts were not significant [14].

Most previous studies on diet and cancers have been performed in Western countries, and few studies have been done in the Middle East, where people’s dietary patterns are likely to be different from western nations due to geographic differences, socioeconomic status, and food habits, preferences, and food availability [7]. People in the Middle East consume high amounts of refined grains, hydrogenated fats, and low amounts of fruits and vegetables and dietary fiber [15]. The association between some components of such dietary patterns with glioma has earlier been reported [16, 17]. In addition, because of the nutritional transition in this region consumption of processed foods is growing in Iran and given the high nitrate content of these products, they might contribute to the increasing prevalence of cancers [18]. However, there is no information assessing the association between whole dietary patterns and risk of glioma in this population. Therefore, we aimed to explore the association between posteriori dietary patterns and risk of glioma in a group of Iranian adults.

## Methods

### Participants

In the current case–control investigation, we recruited patients with newly-diagnosed glioma from three hospitals affiliated to Shahid Beheshti University of Medical Sciences including Loghman Hakim, Imam Hussein and Shohadaye Tajrish hospital in Tehran, Iran. This process was done from November 2009 to September 2011. Selection of cases was done based on convenience-sampling method, in which any individual diagnosed with this type of cancer in the past month was enrolled, albeit after his/her agreement. With 80% study power, type I error of 0·05 and desired Confidence Interval (CI) of 0·95, we needed a minimum of 115 cases and at least 230 controls without glioma. However, we recruited 128 cases and 256 controls, all from a referral hospital for glioma in Tehran, Iran. In brief, out of 235 newly diagnosed patients with glioma that were referred by the pathologist, 40 cases did not meet the inclusion criteria, 15 cases were severely sick and were not able to be interviewed, 22 cases denied participation and 30 cases did not provide adequate information about study variables (lack of accurate and complete answers to the questionnaires). Therefore, 128 patients (75 males and 53 females) with glioma were included. Cases were individuals with confirmed pathological glioma (morphological codes ICD-O-2 9380–9481) during the past month. To get included in the study, they had to be between 20 and 75 years old. We did not include individuals with a prior history of chemotherapy or radiotherapy (due to cancer) and individuals with a confirmed pathological history of any type of cancer (except glioma). Participants in the control group were between 20 and 75 years who were hospitalized in other wards (orthopedic or surgical wards) of the same hospital or referred to the same clinic affiliated to Shahid Beheshti University of Medical Sciences. In addition, individuals with chronic digestive disorders, liver disease, diabetes and other metabolic disorders, immune system disorders and those who followed a special diet (such as a weight loss diet) were not included in the study. Cases and controls were matched in terms of age and sex. All cases and controls provided their informed written consent. This study has been ethically approved by the Research Council of Food Security Research Center, Isfahan University of Medical Sciences, Isfahan, Iran.

### Dietary assessment

A Willett-format-validated 126-item semi-quantitative Food Frequency Questionnaire (FFQ) was used to obtain information on usual dietary intakes of study participants. Validity and reliability of the FFQ have earlier been examined and the findings revealed a reasonable assessment of long-term dietary intakes [19]. The questionnaire was consisted of 126 food items with standard portion sizes commonly consumed by this population. Trained interviewers, who were experienced in completing such questionnaires, filled the FFQ through face-to-face interviews. Interviews were also conducted in the presence of individuals who were involved in the preparation and cooking of foods. Cases were asked to report their usual dietary intakes during the year before glioma diagnosis and controls were asked to report their usual dietary intakes during the year prior to interview based on a given serving of food on a daily, weekly or monthly basis. All reported consumption frequencies were converted to daily grams using Iranian household measures [20]. Then, daily energy and nutrient intakes were determined using the US Department of Agriculture food composition database [21], which was modified for Iranian foods. The interviewer was completely unaware of the research hypotheses, but was aware of the participants' condition (disease-related).

### Assessment of dietary patterns

In the current study, we used principal component analysis to identify major dietary patterns. First, in order to reduce data complexity, the 126 food items were classified into 36 previously defined groups (Table 1). This classification was done based on foods’ similarities, nutrient composition or their kitchen usages, and based on previous studies [15]. Some foods (such as egg) that had unique nutritional profile was considered as an individual food group.

**Table 1:** Food grouping used in the dietary pattern analysis

Food groups	Food items
Processed meats	Salted and smoked meat, hamburgers, sausage, cold cuts, other cured meat (like nugget)
Cured fish	Canned tuna fish, smoked and salted fish
Red meats	Beef, lamb, kebab
Organ meats	Beef liver, brain, tongue, kidney
Fish	Fried fish or fish sandwich and other fish broiled or baked
Poultry	Chicken
Eggs	Eggs
Dairy products	Skim, low or high-fat and whole milk, low and high-fat yogurt, chocolate milk, cream and cheese
Citrus fruits and juices	Oranges, tangerine, lemons, grapefruit, orange juice or grapefruit juice, lemon juice
Other fruits and juices	Pomegranates, kiwi, melon, cantaloupe, watermelon, dates, dried fruits, raisins or grapes, pears, peaches, plums, mulberry, cherries, apricots, strawberries, bananas, apples, other fruit juices (except for citrus fruits juices)
Cruciferous vegetables	Cabbage or cauliflower and Brussels sprouts
Yellow vegetables	Carrots, carrot juice
Tomato	Tomato, tomato sauce
High-nitrate vegetables	Beet, turnip, radish, lettuce, spinach, celery, other vegetables (like parsley, coriander, etc.)
Other vegetables	Green beans, raw onions, bell pepper, mixed vegetables, green vegetable, eggplant, squash, cucumber
Legumes	Beans, peas, lentils, soy, split peas, other legumes
Garlic	Garlic
French fries	French fries
Whole grains	Dark breads (Iranian), barley bread, popcorn and corn, wheat germ, bulgur
Refined grains	White breads (lavash, baguettes, tafton), pasta, rice, potatoes
Snacks	Potato chips
Nuts	Walnuts, roasted seeds, mixed nuts (peanuts, almonds, pistachios, hazelnuts)
Olives	Olives, olive oils
Non-healthy oils	Hydrogenated fats, animal fats, butter, cream, mayonnaise
Vegetable oils	Vegetable oils (except for olive oil)
Sugars	Sugars, candies, chocolates, jam and honey, local sweets
Sweets and desserts	Pastries
Ice cream	Ice cream
Soft drinks	Soft drinks
Yogurt drink	Doogh
Tea	Tea
Coffee	Coffee
Condiments	Condiments, fried and cooked onions, fried and cooked garlics
Salt	Salt
Pickles	Pickles
Alcohol	Wine, liquor

Then, principal component analysis was applied, in which orthogonal transformation (varimax rotation) was used as a rotation method to better interpretation of data and obtain independent dietary patterns. To retain a factor as a major dietary pattern, we used eigenvalues and Scree plot. Labeling dietary patterns was done based on our interpretation of data and prior literature. Factor loadings show correlation coefficients between each food group and dietary pattern. Only food groups that had an absolute factor loading ≥ 0.30 were considered as important factors in food patterns and were included in analysis. Food groups with a higher factor loading had a higher contribution in a dietary pattern. The factor score for each pattern was then calculated by summing intakes of food groups weighted by their factor loadings, and each participant received a factor score for each pattern.

### Assessment of other variables

Required information about sex, age, marital status, occupation, living place, education, smoking status, history of allergy, history of hypertension, family history of cancer and glioma and use of supplements were gathered using a pretested questionnaire. A trained dietitian also examined anthropometric indicators and filled physical activity questionnaire. Participants' physical activity over the past year was assessed using the International Physical Activity Questionnaire and was reported as Metabolic Equivalents (MET)-h/week. Weight was measured in light clothing without shoes by using a digital scale to the nearest 500 g. Height was measured in a standing position with normal shoulders position using a tape measure to the nearest 0/5 cm and BMI was calculated as weight divided by height (m2). Farmers were considered as having a high-risk job [22]. High-risk residential areas were defined as living close to electromagnetic fields, cell phone and broadcast antennas during the last 10 years [23]. In addition, use of fried foods canned foods and barbecue at least twice per week was considered as risk factors.

### Statistical analysis

First, we classified the obtained dietary patterns from factor analysis into quartiles to investigate the associations. One-way analysis of variance with Tukey’s post hoc comparisons was used to assess differences in general characteristics across quartiles for quantity variables. Chi-square test was used to evaluate the distribution of participants in terms of categorical variables. To determine the association between dietary patterns and odds of glioma, we used multivariable logistic regression analysis, in which the effect of sex, age, energy intake, physical activity, education, cigarette smoking, history of allergy, history of hypertension, family history of cancer, family history of glioma, marital status, Living place, high-risk residential area, frequent fried food intake, frequent use of barbecue, frequent use of canned foods and finally BMI was taken into account in different models. In all these analyses, the first quartile of dietary pattern scores was considered a reference. All statistical analyses were done by SPSS (version 16).

## Results

Using factor analysis, we identified 3 major dietary patterns. Based on food loaded in these dietary patterns, they were labeled as: high protein dietary pattern (which was highly loaded with poultry, cured fish, processed meats, fish, eggs, sweet and desserts, coffee, nuts, olives and yellow vegetables), vegetarian dietary pattern (high in other vegetables, other fruits and juices, high-nitrate vegetables, citrus fruits and juices, legumes, condiments, cruciferous vegetables, pickles, tomato and yogurt drink) and western dietary pattern (high in non-healthy oils, soft drinks, refined grains, alcohol, vegetable oils, sugar and red meats). The factor-loading matrixes for these dietary patterns are shown in Table 2.

**Table 2:** Factor-loading matrix for major dietary patterns^a^

Food groups	Dietary patterns		
	High protein dietary pattern	Vegetarian dietary pattern	Western dietary pattern
Processed meats	0.53	-	0.37
Cured fish	0.56	-	-
Red meats	-	-	0.41
Organ meats	-	-	-
Fish	0.51	-	-
Poultry	0.67	-	-
Eggs	0.51	-	0.36
Dairy products	0.32	-	-
Citrus fruits and juices	0.35	0.52	-
Other fruits and juices	-	0.60	-
Cruciferous vegetables	-	0.50	-
Yellow vegetables	0.41	0.33	-
Tomato	-	0.48	-
High-nitrate vegetables	0.39	0.55	-
Other vegetables	-	0.71	-
Legumes	-	0.51	-
Garlic	-	0.32	-
French fries	-	-	0.32
Whole grains	-	-	-
Refined grains	-	-	0.52
Snacks	-	-	0.35
Nuts	0.43	0.38	-
Olives	0.42	-	-
Non-healthy oils	0.30	-	0.60
Vegetable oils	0.36	-	0.46
Sugars	-	-	0.43
Sweets and desserts	0.50	-	-
Ice cream	-	-	-
Soft drinks	-	-	0.53
Yogurt drink	-	0.42	-
Tea	-	-	-
Coffee	0.44	-	-
Condiments	-	0.51	-
Salt	-	-	-
Pickles	-	0.50	-
Alcohol	-	-	0.47
Percentage of variance explained (%)	0.101	0.100	0.081

^a^Values < 0.30 were excluded for simplicity

Characteristics of cases and controls across quartiles of major dietary patterns’ scores are shown in Table 3. Among controls, greater adherence to the high protein dietary pattern was associated with a younger age and less physical activity. They were also less likely to be married and illiterate and more likely to take supplements than those in the lowest quartile. Controls in the top quartile of vegetarian dietary pattern were less likely to smoke and more likely to be married than those in the bottom quintile. Consumption of western dietary pattern among controls was associated with younger age. In addition, controls in the highest quartile had higher weight and were more likely to be men and smoker than those in the lowest quartile. Also, more adherence to the western dietary pattern was associated with more fried foods intake and barbecue use.

**Table 3:** Characteristics of study participants by quartile (Q) categories of dietary pattern scores

	High protein dietary pattern score					Vegetarian dietary pattern score					Western dietary pattern score				
	Q1 (lowest) (*n* = 96)	Q2 (*n* = 96)	Q3 (*n* = 96)	Q4 (highest) (*n* = 96)	*P*-value	Q1 (lowest) (*n* = 96)	Q2 (*n* = 96)	Q3 (*n* = 96)	Q4 (highest) (*n* = 96)	*P*-value	Q1 (lowest) (*n* = 96)	Q2 (*n* = 96)	Q3 (*n* = 96)	Q4 (highest) (*n* = 96)	*P*-value
**Controls**															
Age (y)^a^	42.8 (10.3)	45.6 (13.8)	43.8 (14.3)	38.7 (13.3)	0.022	42.6 (15.5)	43.2 (13.7)	44.8 (12.4)	40.7 (12.1)	0.336	47.2 (12.1)	45.4 (13.3)	43.6 (13.0)	32.9 (10.3)	< 0.001
BMI (kg/m2)^a^	26.5 (3.59)	26.5 (4.3)	25.3 (3.5)	26.2 (3.9)	0.279	25.9 (4.8)	25.9 (3.8)	26.1 (3.6)	26.5 (3.4)	0.768	26.2 (3.9)	25.8 (3.7)	26.5 93.9)	26.0 (3.9)	0.726
Weight (kg)^a^	73.2 (9.3)	71.8 (12.2)	70.1 (11.5)	73.4 (14.3)	0.396	72.8 (11.9)	71.0 (10.5)	71.3 (11.8)	73.0 (13.7)	0.726	67.7 (11.0)	72.4 (12.3)	73.4 (10.9)	75.9 (13.0)	0.001
Physical activity (MET-h/d)^a^	36.3 (5.3)	34.5 (5.8)	33.1 (4.8)	31.9 (5.1)	< 0.001	33.7 (5.9)	32.6 (5.5)	34.0 (4.4)	34.8 (5.9)	0.156	33.6 (5.1)	33.6 (6.3)	34.6 (5.4)	33.6 (5.2)	0.686
Sex (female) (%)	38.5	47.9	39.4	39.4	0.652	32.7	34.4	44.8	50.7	0.121	67.6	39.1	29.5	23.2	< 0.001
Family history of cancers (%)	25.0	38.0	30.3	39.4	0.301	40.4	31.1	29.9	34.7	0.639	37.8	31.3	23.0	42.9	0.112
Family history of glioma (%)	0.0	5.6	12.1	1.5	0.010	9.6	4.9	0.0	6.7	0.103	4.1	4.7	6.6	5.4	0.926
History of allergy (%)	30.8	26.8	25.8	34.8	0.648	26.9	27.9	29.9	32.0	0.925	32.4	37.5	21.3	25.0	0.187
History of hypertension (%)	3.8	7.0	6.1	3.0	0.698	1.9	8.2	6.0	4.0	0.460	12.2	1.6	3.3	1.8	0.012
Living place (Tehran) (%)	53.8	52.1	57.6	68.2	0.241	48.1	52.5	62.7	65.3	0.162	66.2	57.8	47.5	58.9	0.186
Cigarette smoking (%)	26.9	25.4	28.8	18.2	0.521	40.4	27.9	20.9	14.7	0.008	8.1	31.3	32.8	30.4	0.001
Married (%)	98.1	84.5	74.2	66.7	< 0.001	55.8	72.1	94.0	90.7	< 0.001	87.8	78.1	80.3	71.4	0.133
Years of education ≤ 12 (%)	100.0	90.1	74.2	71.2	< 0.001	78.8	78.7	91.0	82.7	0.208	77.0	82.8	93.4	80.4	0.075
High-risk job^b^ (%)	7.7	2.8	0.0	1.5	0.072	0.0	4.9	0.0	5.3	0.097	1.4	4.7	4.9	0.0	0.251
High-risk residential areas^c^ (%)	15.4	22.5	28.8	18.2	0.296	11.5	26.2	23.9	22.7	0.247	18.9	17.2	23.0	28.6	0.436
Taking vitamin supplements (%)	7.7	12.7	13.6	27.3	0.019	17.3	16.4	13.4	16.0	0.942	23.0	12.5	13.1	12.5	0.241
Frequent fried foods intake^d^ (%)	84.6	80.3	78.8	69.7	0.238	71.2	77	77.6	84.0	0.386	66.2	79.7	82.0	87.5	0.022
Frequent canned foods intake^d^ (%)	0.0	4.2	7.6	10.6	0.086	1.9	3.3	9.0	8.0	0.265	2.7	7.8	4.9	8.9	0.420
Frequent use of barbecue^d^ (%)	9.6	11.3	18.2	9.1	0.363	15.4	4.9	9.0	18.7	0.068	2.7	7.8	13.1	28.6	< 0.001
**Cases**															
Age (y)^a^	45.7 (15.6)	42.3 (14.7)	45.3 (15.2)	385 (11.3)	0.176	40.1 (15.4)	43.7 (13.5)	46.7 (15.1)	44.5 (13.7)	0.287	45.2 (8.0)	42.2 (16.9)	48.2 (15.6)	38.8 (13.3)	0.041
BMI (kg/m2)^a^	25.1 (4.8)	27.1 (4.3)	27.5 (3.5)	26.2 (3.6)	0.074	25.8 (3.8)	26.8 (3.6)	25.5 (3.8)	27.8 (6.1)	0.199	27.3 (3.8)	25.3 (3.4)	25.8 (4.2)	27.0 (5.0)	0.240
Weight (kg)^a^	70.3 (13.1)	75.9 (16.4)	77.2 (10.6)	78.0 (13.4)	0.061	73.4 (14.0)	75.9 (11.8)	71.2 (10.9)	80.9 (17.3)	0.077	76.7 (9.8)	72.7 (10.3)	71.2 (13.6)	78.7 (16.6)	0.078
Physical activity (MET-h/d)^a^	34.5 (6.5)	35.0 (6.3)	34.3 (6.1)	35.5 (6.7)	0.880	34.8 (6.9)	34.7 (5.5)	34.3 (5.4)	35.6 (7.7)	0.915	35.6 (5.0)	35.8 (8.3)	33.4 (5.0)	34.8 (6.2)	0.420
Sex (female) (%)	48.8	48.0	43.3	24.1	0.170	39.5	48.6	48.3	25.0	0.312	52.4	25.0	54.3	38.5	0.069
Family history of any cancers (%)	23.3	28.0	33.3	48.3	0.156	27.9	37.1	31.0	35.0	0.839	28.6	40.6	22.9	35.9	0.422
Family history of glioma (%)	9.3	32.0	33.3	10.3	0.015	11.6	34.3	17.2	15.0	0.076	28.6	31.3	11.4	12.8	0.093
History of allergy (%)	34.9	28.0	16.7	17.2	0.224	27.9	40.0	13.8	10.0	0.034	23.8	25.0	25.7	25.6	0.999
History of hypertension (%)	22.3	0	6.7	0	0.294	0	2.9	3.4	5	0.609	0	6.3	0	2.6	0.326
Living place (Tehran) (%)	46.5	72.0	46.7	58.6	0.162	55.8	62.9	41.4	55.0	0.387	57.1	43.8	62.9	53.8	0.468
Cigarette smoking (%)	16.3	16.0	10.0	20.7	0.731	18.6	20.0	13.8	5.0	0.463	0	18.8	8.6	28.2	0.018
Married (%)	72.1	92.0	76.7	79.3	0.360	79.1	77.1	79.3	80.0	0.999	90.5	75.0	85.7	69.2	0.306
Years of education ≤ 12 (%)	90.7	96.0	90.0	75.9	0.109	88.4	91.4	79.3	95.0	0.329	81.0	90.6	91.4	87.2	0.653
High-risk job^b^ (%)	16.3	8.0	6.7	6.9	0.455	11.6	14.3	6.9	5.0	0.645	14.3	15.6	8.6	5.1	0.458
High-risk residential areas^c^ (%)	18.6	40.0	16.7	51.7	0.005	37.2	20.0	27.6	35.0	0.384	28.6	25.0	28.6	35.9	0.781
Taking vitamin supplements (%)	4.7	8.0	10.0	10.3	0.790	2.3	8.6	6.9	20.0	0.115	0	3.1	14.3	10.3	0.166
Frequent fried foods intake^d^ (%)	86.0	88.0	96.7	93.1	0.433	93.0	97.1	89.7	75.0	0.051	81.0	87.5	94.3	94.9	0.258
Frequent canned foods intake^d^ (%)	2.3	0	0	20.7	< 0.001	9.3	2.9	0	10.0	0.248	4.8	6.3	8.6	2.6	0.721
Frequent use of barbecue^d^ (%)	9.3	16.0	13.3	27.6	0.209	18.6	11.4	20.7	10.0	0.617	9.5	6.3	14.3	28.2	0.060

*MET* Metabolic equivalents, *BMI* Body mass index

^a^ Values are Mean (standard deviations)

^b^ Farmers were considered as having a high-risk occupation

^c^ Persons who lived in places nearby electromagnetic fields and cell phone and broadcast antennas in the last 10 years were considered as living in high-risk areas

^d^ Persons who used fried foods, canned foods and barbecue at least twice per week were considered as frequent users

Compared to the lowest quartile, cases in the highest quartile of the high protein dietary pattern were more likely to live in high-risk residential areas and consume canned foods. Greater adherence to vegetarian dietary pattern was associated with lower history of allergy. Cases in the top quartile of western dietary pattern were younger and more likely to smoke than those in bottom quartile.

Dietary intakes of cases and controls across quartiles of major dietary patterns’ scores are shown in Table 4. Among controls, greater adherence to high protein dietary pattern was associated with higher intake of energy, dairies, meats, vitamin A, beta-carotene, vitamin E and calcium. In addition, they consumed higher amounts of dietary proteins, fats and cholesterol and lower amounts of non-healthy oils and grains. Following the vegetarian dietary pattern among controls was associated with greater intakes of energy, vegetables, fruits, dairies, non-healthy oils, legumes, vitamin A, beta-carotene, vitamin C and fats and lower intakes of carbohydrates. Controls in the highest quartile of western dietary pattern had higher intakes of energy, grains, carbohydrates and non-healthy oils and lower intake of vitamin E compared to those in the lowest quartile.

**Table 4:** Dietary intakes of study participants by quartile (Q) categories of dietary pattern scores^a^

	High protein dietary pattern score					Vegetarian dietary pattern score					Western dietary pattern score				
	Q1 (lowest) (*n* = 96)	Q2 (*n* = 96)	Q3 (*n* = 96)	Q4 (highest) (*n* = 96)	*P*-value^b^	Q1 (lowest) (*n* = 96)	Q2 (*n* = 96)	Q3 (*n* = 96)	Q4 (highest) (*n* = 96)	*P*-value^b^	Q1 (lowest) (*n* = 96)	Q2 (*n* = 96)	Q3 (*n* = 96)	Q4 (highest) (*n* = 96)	*P*-value^b^
**Controls**															
Total energy	2341.7 (422.3)	2446.3 (588.2)	2644.5 (800.1)	2764.3 (884.5)	0.011	2248.0 (735.7)	2403.2 (576.1)	2526.9 (511.9)	2928.6 (828.2)	< 0.001	2255.3 (480.0)	2406.3 (516.1)	2608.1 (747.3)	3079.4 (870.3)	< 0.001
Vegetables	246.7 (60.4)	263.5 (81.1)	280.1 (73.4)	288.5 (110.2)	0.594	197.1 (44.5)	226.1 (34.0)	274.0 (51.2)	355.5 (87.1)	< 0.001	277.6 (87.4)	262.1 (81.9)	247.2 (67.6)	297.6 (96.3)	0.011
Fruits	334.4 (132.8)	351.6 (111.6)	362.0 (119.7)	400.8 (131.8)	0.431	248.8 (76.4)	324.4 (93.7)	368.5 (80.6)	470.4 (121.2)	< 0.001	371.8 (125.3)	334.4 (122.1)	339.7 (100.9)	411.9 (138.5)	0.121
Dairies	333.6 (136.4)	342.8 (116.4)	385.8 (133.0)	425.9 (125.2)	0.012	317.9 (126.7)	364.1 (121.0)	357.5 (112.2)	434.1 (138.5)	0.011	387.4 (129.2)	345.7 (104.2)	358.1 (117.0)	403.8 (168.1)	0.049
grains	611.5 (163.9)	609.0 (172.7)	605.9 (202.1)	590.7 (226.5)	< 0.001	573.1 (149.6)	587.9 (190.4)	609.0 (168.9)	634.0 (236.2)	0.669	473.2 (138.5)	590.6 (153.2)	635.0 (147.0)	758.3 (218.3)	< 0.001
Meats	55.7 (15.9)	69.2 (19.9)	77.9 (21.7)	104.1 (71.1)	< 0.001	75.3 (22.2)	72.2 (26.6)	72.1 (21.3)	89.0 (70.2)	0.393	63.6 (20.1)	79.7 (20.0)	72.0 (25.2)	100.4 (78.3)	0.086
Non-healthy oils	30.5 (16.7)	16.3 (14.7)	13.9 (11.6)	12.6 (10.4)	< 0.001	9.7 (8.6)	17.8 (15.6)	21.4 (15.6)	19.6 (15.3)	< 0.001	7.1 (6.2)	13.2 (10.6)	21.9 (13.7)	31.9 (15.6)	< 0.001
Legumes	37.1 (15.3)	40.3 (14.6)	42.4 (20.0)	43.8 (23.2)	0.901	27.6 (10.1)	34.3 (13.9)	41.2 (13.1)	55.9 (20.8)	< 0.001	42.4 (19.7)	40.4 (19.0)	37.3 (16.4)	44.2 (19.1)	0.201
Proteins	86.1 (17.5)	91.7 (20.1)	97.5 (23.1)	110.5 (43.8)	0.001	85.4 (16.7)	91.0 (21.2)	95.3 (18.9)	111.2 (42.6)	0.532	86.6 (17.8)	93.2 (19.5)	95.4 (20.5)	11.6 (46.9)	0.288
Carbohydrates	393.0 (79.5)	400.3 (110.4)	427.8 (162.4)	421.5 (137.5)	< 0.001	364.1 (157.5)	394.5 (108.8)	411.3 (89.3)	458.1 (136.1)	0.001	345.8 (75.5)	387.9 (92.5)	435.2 (155.9)	499.0 (130.6)	< 0.001
Fats	53.3 (13.3)	61.1 (18.5)	67.6 (17.2)	79.8 (25.9)	< 0.001	56.4 (15.9)	58.7 (16.9)	63.3 (17.1)	81.2 (24.2)	0.001	67.5 (20.3)	61.9 (16.3)	61.2 (19.3)	74.0 (28.0)	< 0.001
Cholesterol	197.6 (77.3)	209.1 (71.0)	234.7 (92.3)	292.3 (183.9)	0.009	211.1 (83.2)	198.6 (72.2)	232.1 (75.8)	283.9 (180.6)	0.271	194.4 (71.0)	208.3 (67.9)	243.2 (87.6)	309.8 (197.4)	0.449
Vitamin A	1068.6 (327.3)	1278.9 (416.0)	1479.7 (470.5)	1785.4 (1087.8)	< 0.001	1111.7 (355.9)	1134.2 (363.0)	1382.3 (416.5)	1896.8 (992.4)	< 0.001	1364.6 (597.0)	1416.5 (630.3)	1349.2 (619.6)	1570.1 (952.4)	0.409
Beta-carotene	791.8 (308.2)	942.2 (350.8)	1104.9 (442.2)	1346.5 (987.2)	< 0.001	805.7 (300.5)	817.9 (317.7)	1028.8 (375.2)	1455.3 (908.8)	< 0.001	1045.2 (528.3)	1097.9 (615.0)	987.2 (554.8)	1107.9 (815.3)	0.293
Vitamin E	3.4 (1.6)	4.9 (2.5)	6.3 (2.8)	7.3 (3.0)	< 0.001	5.0 (2.7)	5.5 (3.1)	5.2 (2.6)	6.4 (3.1)	0.479	6.0 (2.8)	5.7 (2.7)	4.9 (2.8)	5.6 (3.4)	< 0.001
Vitamin C	123.1 (43.2)	133.2 (42.8)	163.7 (200.9)	151.3 (59.4)	0.540	96.3 (20.5)	117.5 (25.0)	161.5 (197.3)	181.9 (54.5)	0.008	139.6 (43.4)	151.8 (205.8)	130.7 (37.9)	154.1 (63.7)	0.223
Calcium	981.5 (226.7)	1034.5 (255.7)	1148.2 (296.2)	1292.2 (380.3)	< 0.001	967.4 (258.1)	1073.3 (260.3)	1112.6 (249.50	1269.7 (389.5)	0.279	1077.5 (254.9)	1039.9 (273.9)	1092.9 (223.1)	1296.3 (448.1)	0.084
**Cases**															
Total energy	2450.9 (501.8)	2503.7 (565.2)	2653.6 (508.7)	2773.4 (648.8)	0.217	2394.8 9579.00	2617.9 (481.2)	2594.1 (409.9)	2909.2 (699.2)	0.002	2509.1 (441.2)	2414.8 (493.3)	2439.9 (538.3)	2888.7 (584.3)	0.001
Vegetables	240.1 (84.0)	272.85 (98.4)	256.6 (61.4)	265.1 (71.1)	0.424	188.3 (48.6)	249.3 (35.6)	285.9 (42.1)	370.8 (79.6)	< 0.001	250.0 (69.8)	247.6 (78.8)	251.4 (72.7)	270.7 (91.6)	0.982
Fruits	324.6 (105.1)	327.8 (114.3)	318.5 (60.7)	344.0 (112.6)	0.712	269.2 (71.3)	327.9 (70.1)	357.4 (89.7)	413.3 9 (130.6)	< 0.001	310.9 (67.9)	320.6 (117.4)	305.8 (91.3)	363.9 (98.8)	0.291
Dairies	305.2 (125.4)	284.4 (88.8)	339.3 (98.3)	364.9 (132.0)	0.101	275.9 (117.2)	337.3 (98.6)	332.3 (102.9)	384.3 (134.3)	0.063	322.0 (102.9)	330.8 (121.4)	318.9 (130.5)	320.0 (112.1)	0.440
grains	733.3 (216.4)	724.1 (260.6)	698.6 (186.7)	687.9 (219.5)	< 0.001	689.2 (227.9)	721.1 (222.9)	682.1 (178.6)	794.2 (222.0)	0.237	667.9 (220.1)	660.5 (184.5)	665.2 (193.2)	831.9 (217.2)	0.322
Meats	59.8 (24.8)	72.0 (22.5)	83.9 (22.6)	119.6 (41.5)	< 0.001	84.5 (48.6)	73.9 (25.5)	84.3 (23.7)	84.6 (36.9)	0.223	82.9 (29.8)	70.7 (31.1)	78.8 (35.1)	92.2 (42.3)	0.153
Non-healthy oils	38.1 (12.6)	23.1 (15.8)	17.1 (17.6)	12.8 (10.7)	< 0.001	22.4 (15.7)	24.5 (18.8)	26.6 (19.5)	25.3 (16.5)	0.899	7.1 (8.3)	16.1 (12.1)	24.5 (14.9)	40.4 (13.1)	< 0.001
Legumes	37.0 (25.5)	35.6 (21.9)	33.4 (17.4)	41.2 (20.3)	0.557	27.1 (17.4)	31.9 (17.0)	48.4 (21.3)	49.7 (26.1	< 0.001	42.5 (19.1)	28.9 (17.6)	34.0 (21.9)	42.8 (24.40	0.124
Proteins	88.5 (15.4)	95.8 (19.00	102.4 (22.1)	110.9 (24.8)	< 0.001	91.7 (21.2)	97.9 (21.0)	100.8 (19.1)	109.9 (23.7)	0.742	106.2 (22.6)	91.2 (17.2)	92.4 (23.6)	105.4 (19.8)	< 0.001
Carbohydrates	426.9 (98.1)	413.7 (100.3)	431.3 (96.5)	427.2 (117.7)	< 0.001	386.8 (96.7)	434.8 (99.5)	429.0 (74.5)	486.7 (123.1)	0.708	401.0 (94.1)	390.1 (81.4)	401.9 (97.0)	488.6 (99.7)	0.017
Fats	50.1 (11.8)	59.3 (18.6)	65.5 (15.5)	77.7 (18.1)	< 0.001	59.7 (22.0)	62.0 (15.2)	60.6 (13.6)	68.1 (22.3)	0.941	64.0 (11.8)	60.9 (20.6)	58.1 (15.8)	64.8 (22.0)	0.046
Cholesterol	177.2 (76.9)	237.5 (93.2)	262.7 (86.5)	364.1 (210.7)	< 0.001	272.9 (176.1)	240.4 (137.2)	244.1 (98.2)	238.5 (123.8)	0.055	267.3 (194.7)	215.8 (66.6)	228.2 (94.1)	294.6 (177.4)	0.385
Vitamin A	1168.7 (548.5)	1297.6 (466.5)	1311.4 (388.7)	1666.1 (787.6)	0.017	1010.1 (468.2)	1327.1 (394.4)	1694.6 (566.4)	1566.4 (767.4)	< 0.001	1410.8 (618.7)	1215.9 (474.6)	1220.5 (512.5)	1515.5 (691.2)	0.504
Beta-carotene	869.9 (422.6)	971.0 (433.2)	994.0 (353.2)	1211.2 (794.2)	0.091	671.2 (362.9)	1003.4 (372.0)	1330.9 (470.1)	1202.5 (73,401)	< 0.001	1140.6 (624.0)	894.9 (411.5)	911.8 (443.9)	1080.0 (613.5)	0.399
Vitamin E	3.9 (1.9)	4.9 (2.7)	5.7 (2.7)	6.2 (2.5)	0.003	4.1 (2.4)	4.8 (2.3)	5.8 (2.4)	6.3 (2.8)	0.045	5.6 (2.1)	4.6 (2.8)	4.9 (2.3)	5.3 (2.7)	0.311
Vitamin C	114.4 (32.9)	127.2 (47.1)	138.2 (99.9)	129.8 (36.80	0.492	95.6 (22.0)	137.7 (91.2)	137.7 (31.5)	154.2 (44.0)	0.006	118.9 (26.3)	117.4 (43.9)	115.9 (30.1)	146.1 (89.6)	0.142
Calcium	944.5 (230.3)	984.1 (250.0)	1076.7 (233.3)	1109.6 (310.7)	0.362	898.7 (251.60	1039.9 (240.8)	1056.0 (180.2)	1201.4 (302.7)	0.024	950.8 (202.5)	1028.2 (266.7)	966.5 (269.8)	1084.4 (272.1)	0.132

^a^ Values are Mean (standard deviations)

^b^ Obtained from ANOVA or Chi-square test. *P*-value for dietary intakes were adjusted for age, sex and energy using ANCOVA

Looking at dietary intake among cases, we observed that adherence to a high protein dietary pattern was associated with a high consumption of meats, vitamin A, vitamin E, proteins, fats and cholesterol, and low consumption of non-healthy oils and grains. Cases in the top quartile of vegetarian dietary pattern had significantly higher intakes of energy, vegetables, fruits, legumes, carbohydrates, vitamin E, vitamin C and calcium. Comparing extreme quartiles, we found that greater adherence to the western dietary pattern among cases was associated with higher energy, carbohydrates and non-healthy oils intake.

**Fig. 1:**
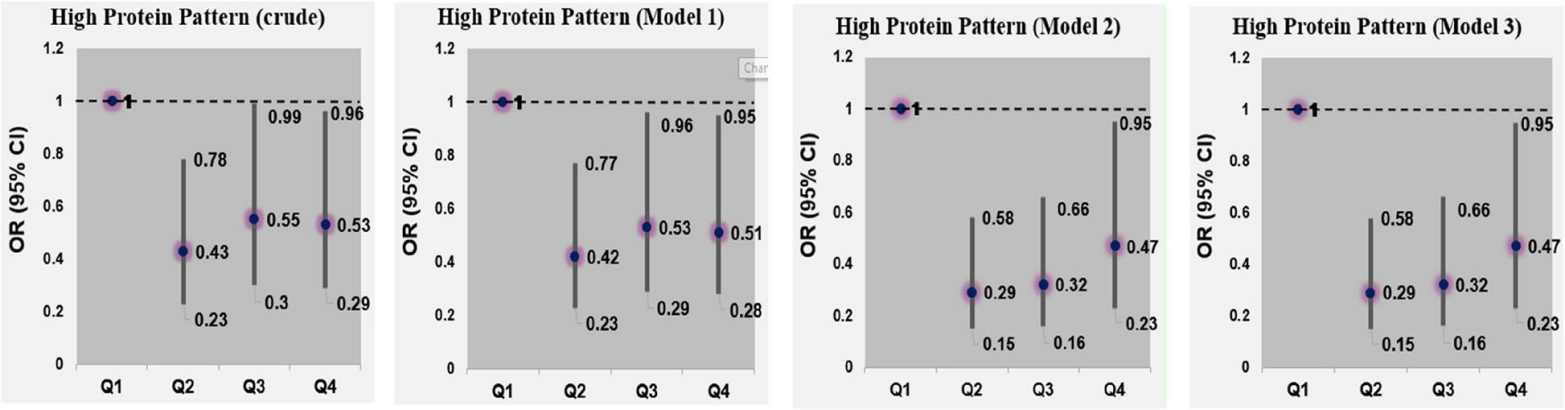
Multivariable-adjusted odds ratios (95% CIs) for glioma across quartile (Q) categories of High protein dietary pattern scores

**Fig. 2:**
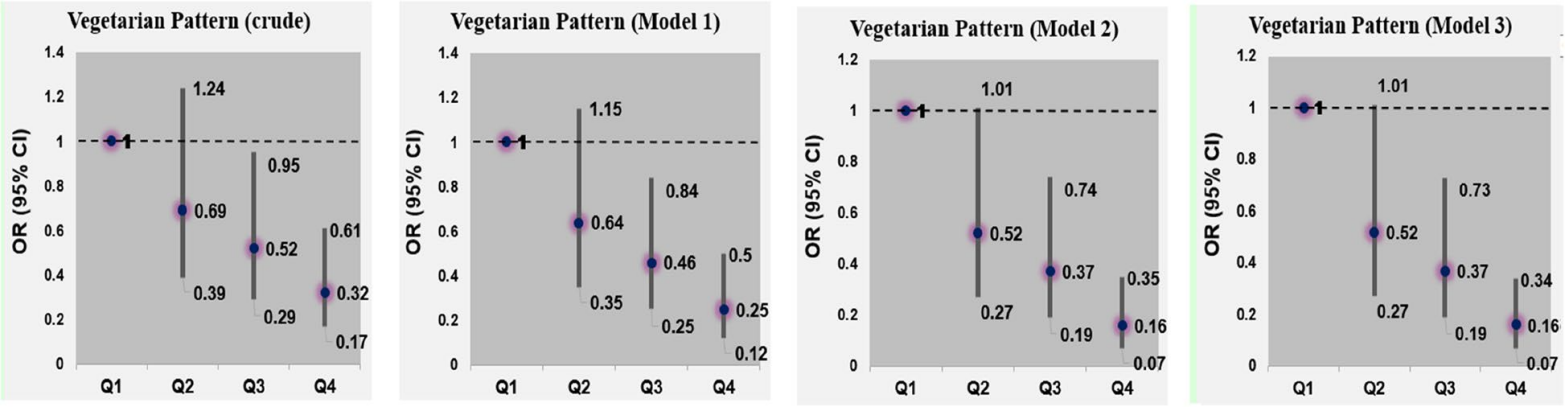
Multivariable-adjusted odds ratios (95% CIs) for glioma across quartile (Q) categories of Vegetarian dietary pattern scores

**Fig. 3:**
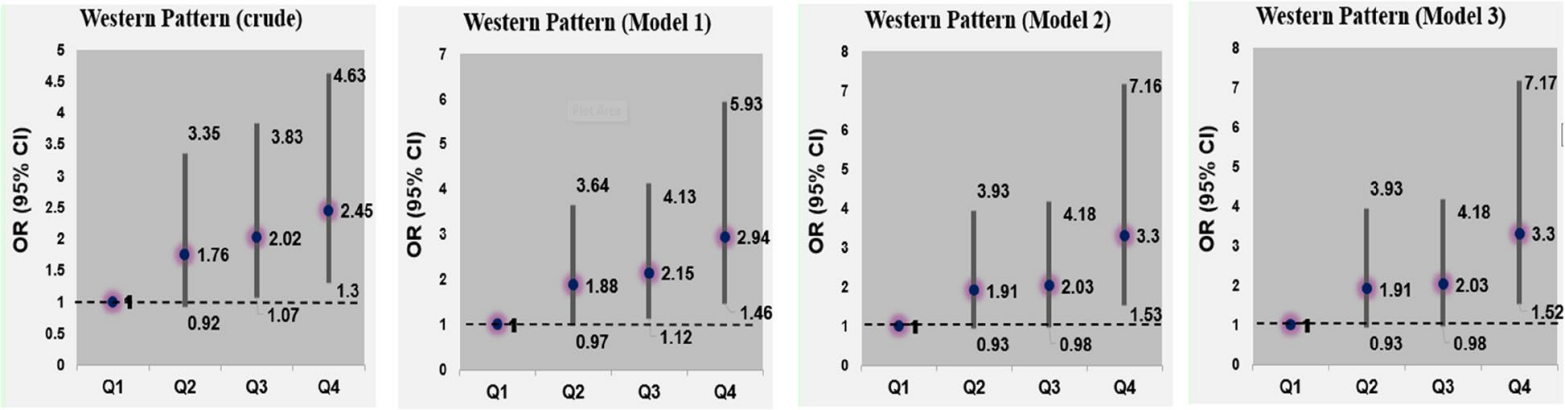
Multivariable-adjusted odds ratios (95% CIs) for glioma across quartile (Q) categories of Western dietary pattern scores

Adjusted odds ratios (ORs) and their 95% CIs for glioma across quartile categories of major dietary patterns’ scores are shown in Table 5. Adherence to the high protein dietary pattern was inversely associated with the risk of glioma (OR: 0.47; 95% CI: 0.23, 0.95) after controlling for several potential covariates. Additional adjustment for BMI had no significant effect on this association (Fig. 1). Consumption of vegetarian dietary pattern was also associated with a reduced risk of glioma; such that those in the highest quartile of this dietary pattern were 84% less likely to have glioma than those in the lowest quartile (OR: 0.16; 95% CI: 0.07, 0.34) (Fig. 2). Greater adherence to western dietary pattern was associated with a greater chance of glioma (OR: 3.30; 95% CI: 1.53, 7.16). This association remained significant even after further adjusting for BMI (OR: 3.30; 95% CI: 1.52, 7.17) (Fig. 3).

**Table 5:** Multivariable-adjusted odds ratios (95% CIs) for glioma across quartile (Q) categories of dietary pattern scores

	High Protein pattern score					Vegetarian pattern score					Western pattern score				
	Q1 (*n* = 96)	Q2 (*n* = 96)	Q3 (*n* = 96)	Q4 (*n* = 96)	P for trend	Q1 (*n* = 96)	Q2 (*n* = 96)	Q3 (*n* = 96)	Q4 (*n* = 96)	P for trend	Q1 (*n* = 96)	Q2 (*n* = 96)	Q3 (*n* = 96)	Q4 (*n* = 96)	P for trend
Crude model	1.00	0.43 (0.23, 0.78)	0.55 (0.30, 0.99)	0.53 (0.29, 0.96)	0.073	1.00	0.69 (0.39, 1.24)	0.52 (0.29, 0.95)	0.32 (0.17, 0.61)	< 0.001	1.00	1.76 (0.92, 3.35)	2.02 (1.07, 3.83)	2.45 (1.30, 4.63)	0.006
Model 1^a^	1.00	0.42 (0.23, 0.77)	0.53 (0.29, 0.96)	0.51 (0.28, 0.95)	0.059	1.00	0.64 (0.35, 1.15)	0.46 (0.25, 0.84)	0.25 (0.12, 0.50)	< 0.001	1.00	1.88 (0.97, 3.64)	2.15 (1.12, 4.13)	2.94 (1.46, 5.93)	0.003
Model 2^b^	1.00	0.29 (0.15, 0.58)	0.32 (0.16, 0.66)	0.47 (0.23, 0.95)	0.039	1.00	0.52 (0.27, 1.01)	0.37 (0.19, 0.74)	0.16 (0.07, 0.35)	< 0.001	1.00	1.91 (0.93, 3.93)	2.03 (0.98, 4.18)	3.30 (1.53, 7.16)	0.004
Model 3^c^	1.00	0.29 (0.15, 0.58)	0.32 (0.16, 0.66)	0.47 (0.23, 0.95)	0.039	1.00	0.52 (0.27, 1.01)	0.37 (0.19, 0.73)	0.16 (0.07, 0.34)	< 0.001	1.00	1.91 (0.93, 3.93)	2.03 (0.98, 4.18)	3.30 (1.52, 7.17)	0.004

^a^Adjusted for sex (male/female), age (continuous) and energy intake (kcal/day)

^b^Further adjusted for physical activity (continues), education (university graduated/nonuniversity education), cigarette smoking (smoker/nonsmoker), history of allergy (yes/no), history of hypertension (yes/no), family history of cancer (yes/no), family history of glioma (yes/no), marital status (married/single/divorced), Living place (Tehran/other cities), high-risk residential area (yes/no), frequent fried food intake (yes/no), frequent use of barbecue (yes/no) and frequent use of canned foods (yes/no)

^c^Additionally adjusted for BMI (continues)

## Discussion

In this case–control study, we found that greater adherence to vegetarian dietary pattern and high- protein dietary pattern was associated with decreased risk of glioma, while consumption of western dietary pattern was associated with a higher risk of glioma. To our knowledge, this study was the first to examine the relationship between major dietary patterns and risk of glioma in the Middle East.

According to our findings, greater adherence to high protein dietary pattern (which was highly loaded with poultry, cured fish, processed meats, fish, eggs, sweet and desserts, coffee, nuts, olives and yellow vegetables) was associated with 53% decreased risk of glioma. Our previous study in this population revealed that consumption of low carbohydrate diet (low in carbohydrates and high in protein and fat), that is nearly similar to high protein dietary pattern was associated with a reduced risk of glioma [24]. Our findings are mostly consistent with other studies that have examined the association between components of high protein dietary pattern separately with glioma risk [25–28]. Although it seems that the consumption of processed meat may be associated with an increased risk of cancers, the results of a meta-analysis showed that consumption of processed red meat was not generally associated with the risk of glioma in case–control or cohort studies [28]. In addition, some components of high protein dietary pattern may have protective effects against glioma. For instance, findings from meta-analysis studies revealed that consumption of fresh fish and coffee has been associated with reduced risk of glioma [25, 27]. In addition, eggs and nuts as components of high protein dietary pattern can also explain the favorable association of this dietary pattern with glioma [26, 29].

In the current study, we found a significant association between vegetarian dietary pattern and risk of glioma; so that greater adherence to vegetarian dietary pattern was associated with a decreased risk of glioma. Adherence to plant-based dietary pattern and also healthy plant-based dietary pattern was also associated with a reduced risk of glioma in earlier studies [30]. In addition, our finding in previous study showed an inverse association between consumption of legumes and nuts and risk of glioma [26]. In contrast, a combined analysis of 3 large prospective studies in the UK and US showed weak evidence for increased risk of glioma by greater adherence to healthy dietary patterns (DASH diet, alternate Mediterranean diet and Alternative Healthy Eating Index). However, finding of that study was attenuated toward the null after excluding the first 5 years of follow-up [13]. Conflicting findings may be explained by differences in study design, study population, dietary assessment methods, as well as different dietary components of the patterns. Overall, given the nature of observational studies, our results need to be confirmed by more comprehensive prospective studies in future.

We found a significant positive association between western dietary pattern (high in non-healthy oils, soft drinks, refined grains, alcohol, vegetable oils, sugar and red meats) and risk of glioma. No other earlier study reported a positive association between western dietary pattern and risk of glioma. However, components of western dietary pattern have been linked with elevated risk of glioma in previous studies. For example, our previous investigation in this population showed a significant positive association between consumption of refined grains and the risk of glioma [17]. In addition, high dietary glycemic index, which is a characteristic of western dietary patterns, has also earlier been shown with elevated risk of glioma [31]. Unlike our findings, some studies reached opposite findings. In a case–control study, an inverse association was reported between a dietary pattern so-called as "snacks" and risk of glioma. That dietary pattern was high in snacks, candy, cookies and cakes, dairy products, ham, sauces, beef, and soft drinks, which were nearly similar to foods loaded in our western dietary pattern [12]. The opposite finings might be explained by several factors: Albuquerque et al. questioned their adult participants about their remote dietary intake during adolescence. Remembering remote dietary intakes and the adherent biases might affect their findings. In addition, discrepancy in the components of dietary patterns between the two studies might provide some other explanations for these different findings.

The protective associations of a high protein dietary pattern against glioma might be attributed to the beneficial components of this pattern. Unsaturated fatty acids in poultry and fish can provide some reasons [32]. Fish is a primary source of omega-3 fatty acids, which can in turn inhibit biosynthesis of eicosanoids derived from arachidonic acid, decrease inflammation, inhibit mutations, and enhance cell apoptosis [33, 34]. In addition, in a previous study we found that a higher intake of polyunsaturated fatty acids was associated with a reduced risk of glioma [35]. Eggs in this dietary pattern as a food source of cholesterol can provide the cholesterol needed to form and maintain lipid rafts and thus through which might help functioning the brain properly. In addition, since eggs are a good source of protein, lecithin and choline, they can help with antioxidant function, synthesis of neurotransmitters and brain enzymes [36, 37]. Nuts provide essential fatty acids, vitamins (especially vitamin E), minerals, antioxidants and polyphenols that can have antioxidant, anti-inflammatory and apoptotic regulatory effects against tumor cells [38].

Several physiological mechanisms can be regarded for the inverse association between vegetarian dietary pattern and risk of glioma. Its high content of vegetables, fruit, legumes, and nuts, which are originally rich in vitamins, minerals, antioxidants and polyphenols might account for this protective association [39]. These components have been shown to reduce oxidative stress and inflammation through which they might prevent the production of reactive oxygen species [40]. In addition, high amount of fiber and phytochemicals in this pattern can play a key role in prevention of cancer by reducing circulating estrogens and androgens, inflammation, insulin resistance, and IGF-1 concentration [41, 42].

The positive association between western dietary pattern and risk of glioma can be attributed to the components of this pattern, e.g. refined grains, sugar and soft drinks. These components have a high glycemic index that might increase plasma insulin concentrations and IGF-1 levels [43]. The IGF-1 signaling system can stimulate cancer progress by preventing apoptosis and stimulating cell proliferation. IGF-1 is a key mitogenic stimulus for tumor cell growth [44]. In addition, consumption of high glycemic index foods has been associated with a greater risk of inflammation and oxidative stress [45, 46]; these factors can eventually result in glioma [47].

Another issue that should be considered is the relationship between the patients' dietary pattern and progression and severity of glioma. Studies in this area are limited. The results of an animal study showed that a low-calorie and low-protein diet could not be effective in reducing the progression of glioma [48]. The findings of a cell study showed the effectiveness of ketogenic diet in reducing the growth of glioma [49]. The findings of another study showed that fructose metabolism is increased in glioma cells and this leads to further tumor growth, so a diet containing high amounts of fructose may affect the progression and severity of the disease in patients with glioma [50]. Also, studies have shown the therapeutic effects of some nutrients including vitamin A, vitamin D and conjugated linoleic acid in glioma [51–53]. Further human studies in the future will help clarify the connections.

Other important issue that should be considered is food processing. The method, duration and temperature of processing have an effect on the nutrient content of food. For example, as a result of cooking vegetables such as spinach, broccoli and sweet potato, their iron and zinc content decreases between 10 and 27 percent [54]. On the other hand, cooking vegetables often increases the bioavailability of iron, zinc and calcium [55]. The loss of some vitamins, such as vitamin C and thiamine, increases with high temperature, while vitamin K and niacin are more resistant to heat [54]. Frying can increase the content of trans fatty acids in food [56]. Therefore, the effect of processing method should be considered when examining the relationship between diet and health-related outcomes. In the present study, we adjusted effect of several processing methods, including frying, canning and using barbecue.

This study had several strengths. Our study was the first to examine the relationship between major dietary patterns and risk of glioma in the Middle East. In addition, to achieve an independent association, several confounders were controlled for in our study. Moreover, we recruited newly diagnosed glioma patients, therefore, change in dietary habits of participants was less likely. However, some limitations need to be taken into account. The case–control design of the study with its adherent errors of recall and selection bias is a limitation, then, one cannot confer causality. Due to the case–control nature of the study and the fact that the controls are hospital-based, our study findings cannot be easily generalized to the community. However, participants in the present study were selected from the main hospitals to which all glioma patients in the country were referred to. Thus, participants had different eating habits and socioeconomic status. Among other limitations of this study, it can be mentioned that the data collection of the present study was done in the past. Due to the possibility of change in the dietary pattern of societies so the results should be interpreted with caution. Although, we recruited new cases of glioma, dietary assessment was performed after diagnosis of cancer. Therefore, cases may recall their past diet differently. Complications of the disease may also have affected the patient's diet prior to diagnosis. Another limitation, as with all epidemiological studies, was the use of FFQ, in which misclassification of participants is unavoidable. Although several confounders were controlled for, one cannot exclude the possibility of residual confounding. In present study information regarding the income level of participants has not been collected, and this is one of the limitations of our study. Because the dietary intake of the Middle-Eastern population differs from Western population, generalizability of our findings to other community must be done with caution.

In conclusion, we found a protective association between vegetarian dietary pattern and also high protein dietary pattern and risk of glioma. On the other hand, a positive association was observed between western dietary pattern and odds of glioma. Further studies, especially of a prospective design, are needed to confirm these findings.

## Data Availability

Not applicable.
